# Supplementary material for: Bioavailability and provitamin A activity of neurosporaxanthin in mice
Source: Commun Biol. 2023 Oct 20;6:1068. doi: 10.1038/s42003-023-05446-1 (PMC10589281; doi:10.1038/s42003-023-05446-1)
Supplement: Supplementary file 2 — Description of Additional Supplementary Files [file 42003_2023_5446_MOESM2_ESM.pdf]

Description of Additional Supplementary Files

File name: Supplementary Data
Description: Numerical data for the figures.
